# Cognitive impairment after cerebral ischemia–reperfusion injury: a neuroecosystem perspective

**DOI:** 10.3389/fneur.2026.1912737

**Published:** 2026-09-22

**Authors:** Jiangbo Xie, Tingting Zhang, Jinmei Zhang, Liping Zhang, Tao Huang, Wenhua Bi, Manli Zhao

**Affiliations:** 1 Department of Neurology, Weifang Hospital of Traditional Chinese Medicine, Shandong Second Medical University, Weifang, China; 2 Department of Rehabilitation, Fujian Medical University, Fuzhou, China; 3 Department of Rehabilitation, Weifang Hospital of Traditional Chinese Medicine, Shandong Second Medical University, Weifang, China; 4 Department of Endocrinology, Weifang Hospital of Traditional Chinese Medicine, Shandong Second Medical University, Weifang, China; 5 Clinical Integration of Traditional Chinese and Western Medicine, Shandong Second Medical University, Weifang, China; 6 Department of Neurosurgery, The Second Affiliated Hospital of Guangzhou University of Chinese Medicine, Guangzhou, China

**Keywords:** cerebral ischemia–reperfusion injury, cognitive impairment, ferroptosis, glial cells, immune cells, neuroecosystem, neurovascular unit

## Abstract

Post-stroke cognitive impairment (PSCI) is a common and debilitating neurological complication following cerebral ischemia–reperfusion injury (CIRI). The traditional neuron-centered view cannot fully explain its delayed onset, pathological heterogeneity, and functional plasticity. This review proposes a novel neuroecosystem framework based on the neurovascular-immune-glial (NIG) triad, including a systematic evaluation of the dynamic crosstalk among the neurovascular unit (NVU), neuronal networks, and glial-immune microenvironment. Pathological events, including ferroptosis, neuroinflammation, white matter damage, and gut-brain axis dysfunction, are reinterpreted as manifestations of neuroecosystem collapse, rather than isolated lesions. We further summarize cutting-edge tools, such as spatial omics and *in vivo* dynamic imaging for framework validation, and clarify how therapeutic strategies are shifting from single neuronal protection to overall ecosystem restoration. Finally, we discuss current limitations in knowledge and future research directions, aiming to provide new insights for mechanistic exploration and clinical intervention of PSCI.

## 1 Introduction

Post-stroke cognitive impairment (PSCI), which affects a substantial proportion of survivors of stroke, represents a significant clinical challenge owing to its contributions to long-term disability and diminished quality of life. Traditionally, the understanding of PSCI has been largely neuron-centric, focusing on the direct impact of ischemic injury on neuronal populations and synaptic networks. However, this conventional neuron-centered paradigm has some limitations when attempting to explain the complexity and delayed onset of cognitive deficits following cerebral ischemia–reperfusion injury (CIRI). Emerging evidence has suggested that cognitive function is not solely dependent on neuronal integrity, but is also influenced by the dynamic equilibrium of a complex neuroecosystem, encompassing neurons, glial cells, immune components, and vascular elements. This intricate interplay forms the neurovascular unit (NVU), which orchestrates cerebral homeostasis, neurovascular coupling, and immune surveillance, and is critical for maintaining cognitive function.

Ischemic stroke, characterized by interrupted cerebral blood flow and subsequent reperfusion injury, initiates a cascade of pathophysiological processes that extend beyond neuronal death alone. Reperfusion therapy, although essential for restoring oxygen and nutrient delivery, paradoxically worsens tissue injury through several mechanisms, including oxidative stress, excitotoxicity, ferroptosis, autophagy dysregulation, and neuroinflammation (1, 2). These mechanisms involve multiple cell types and molecular pathways, highlighting the multifaceted nature of ischemia–reperfusion (I/R) injury. Microglial activation and polarization modulate the inflammatory milieu, with M1- and M2-type microglia promoting neurotoxicity and facilitating repair and neuroprotection, respectively, as demonstrated by the modulation of microglial phenotypes by ginsenoside Rb1, leading to cognitive improvement in ischemic models (3). Similarly, astrocytes respond to ischemic injuries by altering blood–brain barrier (BBB) integrity and water homeostasis, thereby influencing edema formation and neuronal survival (4).

The neurovascular-immune-glia (NIG) triad is a conceptual framework uniting these diverse cellular actors. This triad emphasizes the crosstalk between vascular cells (endothelial cells and pericytes), glial populations (astrocytes, microglia, and oligodendrocytes), and immune elements that collectively regulate cerebral blood flow, metabolic support, immune responses, and synaptic plasticity. Disruption of this triad during I/R leads to NVU dysfunction, neuroinflammation, and, ultimately, cognitive decline. For example, microglial-mediated neuroinflammation, driven by inflammasome activation (such as NLRP3), contributes to synaptic dysfunction and neuronal loss, which can be attenuated by electroacupuncture that modulates mitophagy and inflammatory cascades (5). Moreover, alterations in astrocytic aquaporin-4 polarization impair interstitial fluid drainage, thereby exacerbating the accumulation of neurotoxic proteins and inflammatory mediators, as shown by photobiomodulation enhancing extracellular space molecular transport and improving cognition post-stroke (6).

Beyond glial and immune contributions, vascular pathology plays a pivotal role in PSCI pathogenesis. Large vessel atherosclerosis and small vessel disease, including white matter lesions and microinfarcts, compromise cerebral perfusion and BBB integrity, facilitating the neurodegenerative processes (7, 8). For example, carotid atherosclerotic calcification independently predicts PSCI risk, underscoring the importance of vascular health in cognitive outcomes (7). The BBB and associated glia limitans form a critical barrier regulating immune cell infiltration and maintaining central nervous system (CNS) immune privileges. Matrix metalloproteinases (MMPs), such as MMP-3 and MMP-2, modulate glia limitans integrity and extracellular matrix (ECM) remodeling following ischemic injury, thereby influencing neuroinflammation and NVU stability (1, 9).

The immune system’s role extends to systemic and local inflammation, with circulating biomarkers such as C-reactive protein and neurofilament heavy chain reflecting ongoing neurovascular injury and neuronal degeneration linked to cognitive decline (10, 11). Genetic and epigenetic factors, including microRNAs and DNA methylation status, regulate neuroinflammatory pathways and synaptic plasticity, affecting recovery trajectories (2, 12). Notably, synaptic proteins such as SNAP29 have been implicated in presynaptic maintenance, and their dysregulation contributes to PSCI via disrupted synaptic transmission (13).

Given this complexity, advancing PSCI research requires an integrative neuroecosystem perspective transcending the neuron-centric view. This approach recognizes the brain as an ecosystem in which neurons, glia, immune cells, and vascular components interact dynamically within the NIG triad. Therapeutic strategies targeting this triad, including the modulation of microglial polarization, astrocyte function, vascular integrity, and immune responses, hold potential for mitigating PSCI. Interventions such as electroacupuncture, pharmacological agents (such as ginsenoside Rb1 and polydopamine-based nanoparticles), and neuromodulation techniques (such as transcranial direct current stimulation) exemplify this paradigm, aiming to restore neurovascular homeostasis and promote neuroplasticity (3, 14, 15).

In this review, we aimed to synthesize the recent advances in understanding PSCI through the lens of the NIG triad, thereby highlighting the multisystem interactions underpinning cognitive dysfunction after ischemic stroke and reperfusion injury. By integrating molecular-, cellular-, and system-level insights, we propose a novel neuroecosystem framework for PSCI pathogenesis and discuss the emerging therapeutic avenues that leverage this paradigm to improve cognitive outcomes in survivors of stroke (Figure 1).

**Figure 1:**
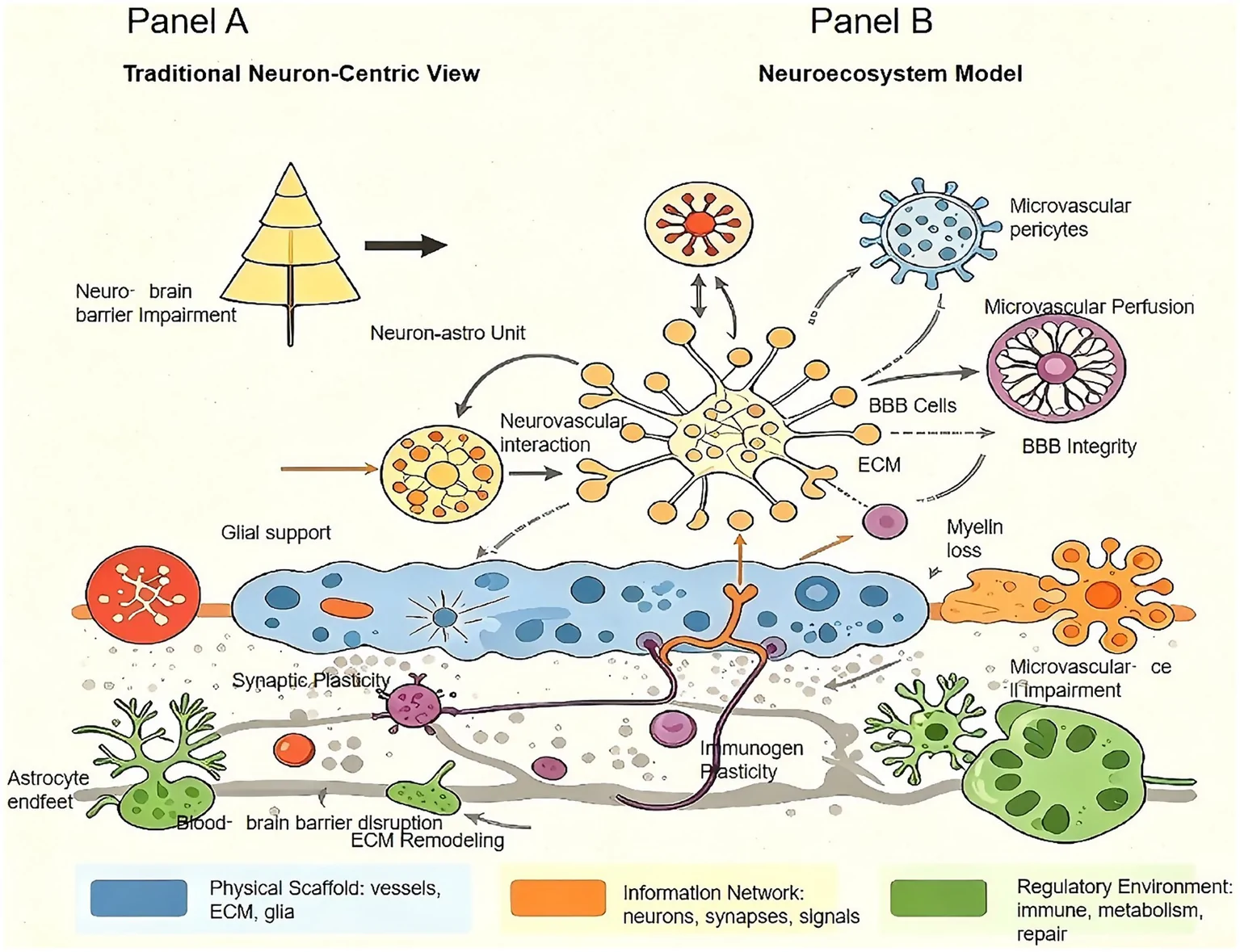
Comparison of the traditional neuron-centric view and the neuroecosystem model for post-stroke cognitive impairment. Schematic illustration comparing the traditional neuron-centric view with the neuroecosystem model. The traditional view emphasizes oversimplified neuronal injury and cognitive impairment, whereas the neuroecosystem model integrates neurons, endothelial cells, pericytes, astrocytes, microglia, oligodendrocytes, circulating immune cells, and ECM remodeling as an interactive system. BBB integrity, synaptic pruning, immune cell-microglia crosstalk, neuroinflammation, myelin loss, and axon guidance collectively contribute to post-stroke cognitive impairment.

However, before proceeding, it is essential to distinguish three interrelated but distinct concepts that are discussed throughout this review. First, CIRI refers to the acute pathophysiological process occurring during the ischemic insult and subsequent reperfusion, encompassing oxidative stress, excitotoxicity, ferroptosis, and acute blood–brain barrier (BBB) disruption. Ischemic stroke denotes the clinical event that includes CIRI in addition to its structural consequences, such as infarct formation, edema, hemorrhagic transformation, and post-stroke complications. Finally, PSCI describes the long-term neurocognitive outcome resulting from cumulative structural, functional, and network damage. While the mechanisms underlying CIRI are primarily addressed in the acute-phase sections, PSCI pathogenesis extends across the subacute and chronic phases, involving white matter degeneration, synaptic remodeling, glymphatic dysfunction, and systemic metabolic influences.

Furthermore, to ensure clarity, the novelty of the neuroecosystem framework warrants explicit clarification relative to existing concepts. The NVU encompasses endothelial cells, pericytes, astrocytic endfeet, basement membrane, and associated neurons, but nevertheless remains a primarily structural concept that does not integrate immune cells, extracellular matrix (ECM) dynamics, or systemic factors. The neuroimmune axis focuses predominantly on microglial activation, astrocytic responses, and peripheral immune cell infiltration, and largely ignores vascular-metabolic coupling and information-processing networks. Uniquely, the neuroecosystem framework proposed herein transcends both by: (1) integrating systemic compartments (the gut-brain axis and metabolic regulation) with local compartments (the NVU and glial-immune microenvironment) into a single dynamic framework, (2) introducing temporal staging of ecosystem collapse and restoration across the acute, subacute, and chronic phases, and (3) mapping stage-specific biomarkers to corresponding therapeutic windows, thereby enabling precision intervention. The NIG triad constitutes the cellular substrate of this ecosystem, comprising vascular cells (endothelial cells and pericytes), immune cells (microglia and infiltrating leukocytes), and glial cells (astrocytes and oligodendrocytes), with microglia serving as a bridge between the immune and glial compartments.

## 2 Composition and functional framework of the neuroecosystem

The NIG triad constitutes the cellular substrate of the neuroecosystem, which manifests functionally as three interdependent layers: the physical scaffold (neurovascular component + ECM), the information network (neuronal populations + synapses + white matter tracts, supported by the glial component), and the regulatory environment (immune + glial components + their secretome). As presented in Figure 1B, these three layers operate in dynamic equilibrium. The following sections detail each functional layer and its corresponding NIG triad components.

### 2.1 Physical scaffold: NVU and ECM

The NVU constitutes a sophisticated physical and functional scaffold which is critical for maintaining the integrity of the BBB and homeostasis of the cerebral microenvironment. Structurally, the NVU is composed of endothelial cells, a basal lamina (basement membrane), astrocytic endfeet, and pericytes, which collectively form the physical foundation of the BBB. Endothelial cells, which line the cerebral microvasculature, are interconnected by tight junction proteins such as claudin-5, and are essential for BBB impermeability and selective molecular transport (16, 17). These endothelial cells are supported by the basal lamina, an ECM rich in collagen IV and fibronectin, which provides mechanical stability and the biochemical cues essential for cellular adhesion and signaling (18, 19). The basal lamina not only anchors endothelial cells but also functions as a substrate for pericytes and astrocyte endfeet, facilitating close cellular interactions within the NVU.

Pericytes embedded within the basal lamina play multifaceted roles, including the regulation of cerebral blood flow, maintenance of BBB integrity, and modulation of inflammatory responses. Their strategic positioning further allows them to communicate bidirectionally with endothelial cells and astrocytes, thus contributing to neurovascular coupling and metabolic support (20, 21). Astrocytic endfeet envelop the microvessels, secreting factors that regulate endothelial tight junctions and ECM remodeling, which are vital for sustaining the selective permeability of the BBB and maintaining the microenvironment required for neuronal function (17, 22). Moreover, astrocytes actively participate in neuroinflammatory processes and repair mechanisms following ischemic injury, underscoring their dualistic roles within the NVU (22).

The ECM surrounding the NVU functions not only as a passive scaffold, but also as a dynamic milieu modulating cellular behavior through biochemical signaling and structural support. Composed of proteins such as collagen IV, laminin, fibronectin, and proteoglycans, the ECM maintains the spatial organization of NVU components and regulates cell migration, differentiation, and survival (23, 24). Ischemic injuries induce significant alterations in ECM composition and structure, including degradation by MMPs, which disrupts NVU integrity and exacerbates BBB breakdown (25, 26).

Conversely, ECM remodeling during the recovery phase supports angiogenesis and neurogenesis, facilitating tissue repair and functional restoration (27). The ECM additionally mediates the signaling pathways influencing NVU cell proliferation and migration, such as those involving tissue inhibitors of metalloproteinases, which balance MMP activity to preserve ECM homeostasis (28).

Functionally, the NVU orchestrates the maintenance of brain microenvironmental homeostasis and metabolic coupling by regulating cerebral blood flow in response to neuronal activity, a process termed neurovascular coupling, ensuring adequate oxygen and nutrient delivery while removing metabolic waste (21, 29). The NVU additionally controls immune cell trafficking and inflammatory responses, with dysfunction leading to neuroinflammation and neurodegeneration (30, 31). Recent advances in *in vitro* 3D models and organ-on-a-chip technologies have recapitulated the complex architecture of the NVU, thereby enabling detailed studies of cell–cell and cell-ECM interactions under physiological and pathological conditions, including ischemic stroke (32, 33). These models further underscore the importance of the physical scaffold provided by the NVU and ECM in preserving BBB function.

In brief, the NVU and its surrounding ECM form an integrated physical scaffold essential for BBB structure and function. Their dynamic interplay ensures the brain’s microenvironment stability, mediates metabolic coupling, and regulates cellular responses to injury. Understanding the structural and functional components of the NVU and ECM is therefore crucial for developing interventions to mitigate neurovascular dysfunction and cognitive impairment following cerebral ischemia and reperfusion injury.

### 2.2 Information networks: neuronal populations, synapses, and white matter fiber tracts

The foundation of cognitive function lies in the intricate architecture of the neuronal populations and their synaptic interconnections, which serve as fundamental units for information processing within the brain. Neurons form complex networks through synapses, thereby enabling the transmission and integration of the electrical and chemical signals essential for cognition. These synaptic connections are not static, instead, they exhibit remarkable plasticity and capacity for remodeling, which are critical for learning, memory, and recovery following injury. The integrity of the white matter fiber tracts, which are composed primarily of myelinated axons, ensures the fidelity of long-distance neural signal conduction between disparate brain regions, thereby supporting coordinated cognitive operations. Recent neuroimaging and histological studies have elucidated the spatial and functional relationships between neuronal elements and their supporting vascular structures, particularly within the white matter. For example, high-resolution multimodal magnetic resonance imaging (MRI) investigations in primate brains have demonstrated that the white matter vasculature aligns closely with axonal geometry and density, reflecting the metabolic demands of axonal branching and synaptic activity, rather than simply the density of overlying gray matter neurons (34). This coupling underscores the metabolic and structural interdependence of the neurons, synapses, and white matter in sustaining cognitive processes.

Microstructural white matter abnormalities have been implicated in various neurological conditions characterized by cognitive deficits. Diffusion kurtosis imaging studies in patients with trigeminal neuralgia have revealed asymmetrical microstructural alterations in the bilateral hemispheres, including compromised integrity of fiber tracts such as the corona radiata and superior longitudinal fasciculus, which correlate with disease duration and pain intensity (35). These findings highlight the vulnerability of the white matter pathways to pathological injuries and their potential roles in cognitive dysfunction. At the molecular level, neuronal cell adhesion molecules (NRCAM) are pivotal in controlling axonal growth, guidance, synaptogenesis, and myelination. Biallelic variants of NRCAM cause neurodevelopmental syndromes characterized by intellectual disability and motor abnormalities, with animal models showing disrupted neurite outgrowth and altered white matter tracts (36). These molecular disruptions translate into impaired formation and maintenance of the neuronal networks critical for cognition.

Beyond neurons, oligodendrocyte precursor cells (OPCs) within the white matter contribute to synaptic gene expression and interact with neurons, thereby influencing neural circuit plasticity. Genetic studies have linked OPC-specific gene expression profiles to psychiatric disorders, indicating that OPCs actively participate in the neural network dysfunctions underlying cognitive and behavioral abnormalities (37). This emerging perspective expands the traditional neuron-centric view by incorporating glial contributions into information networks. White matter dysfunction in Alzheimer’s disease (AD) manifests as decreased functional connectivity and network efficiency within the white matter regions integral to sensory-motor, visuospatial, language, and memory functions. Transcriptomic analyses reveal that these white matter alterations correlate with synaptic function-related gene expression, which is predominantly active in neurons and astrocytes, thereby linking molecular pathology to network-level dysfunction (38).

The plasticity and reorganization capacity of neuronal networks are essential for cognitive recovery following brain injuries, including I/R injury. Experimental models have demonstrated that certain pharmacological interventions, such as levodopa, enhance synaptic plasticity in the hippocampus by increasing synaptic density and the expression of plasticity-related proteins (BDNF, TrkB, PSD95, and drebrin). These changes correlate with improved cognitive outcomes and white matter integrity (39). Conversely, disruptions in inhibitory interneuron maturation following neonatal hypoxic episodes can lead to altered cortical connectivity and long-term cognitive impairments, thus emphasizing the delicate balance between excitatory and inhibitory synaptic networks in cognitive function (40). In addition, large-scale computational modeling of neuronal networks incorporating white matter fiber activation provides insights into therapeutic interventions such as deep brain stimulation, where the modulation of specific pathways can restore network dynamics and improve clinical outcomes (41).

In summary, the information networks underpinning cognition comprise neuronal populations interconnected by synapses and supported by the structural and functional integrity of the white matter fiber tracts. The dynamic plasticity and remodeling of these networks are crucial for maintaining cognitive function and enabling recovery after an injury. Advances in neuroimaging, molecular genetics, and computational modeling have deepened our understanding of these complex systems.

### 2.3 Regulatory environment: glial cells, immune cells, and their secretome

The regulatory environment within the NIG triad is orchestrated by the dynamic interplay among glial, immune, and vascular cells, each of which plays multifaceted roles through direct cellular interactions and the secretion of diverse bioactive molecules. Astrocytes, oligodendrocytes, and microglia, the principal glial cell types, serve not only as neuronal supporters, but also as critical regulators of metabolism and immune responses in the CNS. Specifically, astrocytes maintain BBB integrity by ensheathing endothelial cells with perivascular endfeet and secreting homeostatic soluble factors that preserve vascular stability and modulate immune cell infiltration. In pathological states such as AD, astrocytes exhibit structural alterations, including swollen endfeet and somatic shrinkage, which can compromise vascular integrity at the capillary and arteriole levels, moreover, their skewing toward pro-inflammatory and oxidative phenotypes exacerbates endothelial junction disruption and promotes leukocyte entry into the brain parenchyma (42). Oligodendrocytes, which have been traditionally recognized for axonal myelination, contribute to CNS energy metabolism together with astrocytes and microglia, underscoring their involvement in maintaining neuronal function beyond insulation (43). Microglia, the resident immune cells of the CNS, rapidly respond to ischemic injury by adopting diverse activation states, engaging in the phagocytosis of cellular debris, and releasing extracellular vesicles (EVs) containing proteins, nucleic acids, and metabolites that modulate neuronal survival, neuroinflammation, and vascular repair processes (44). Their secretome includes neurotrophic factors such as insulin-like growth factor 1, which, when dysregulated, contributes to amyloidogenic and vascular dyshomeostasis in neurodegenerative diseases (45).

Peripheral immune cells dynamically interact with the CNS through the BBB, which restricts their entry under physiological conditions, becoming permissive only during inflammation or injury. Neutrophils, the earliest infiltrating immune cells following ischemic stroke, exemplify this dual role—mediating both neuronal apoptosis and repair depending on their activation and differentiation states (46). The crosstalk between peripheral immune and CNS-resident cells is mediated by cytokines and chemokines. For example, in CNS tuberculosis, pro-inflammatory cytokines such as interleukin (IL)-1β, IL-17, and tumor necrosis factor (TNF)-α are all expressed by glial and vascular endothelial cells, thereby contributing to tissue and vascular damage (47). Moreover, gut-licensed β7 + CD4 + T cells have been implicated in retinal ganglion cell degeneration in glaucoma by inducing vascular endothelial expression of mucosal addressin cell adhesion molecule 1, highlighting the systemic immune influence on CNS vascular-immune interactions (48).

Vascular cells, including endothelial cells and pericytes, secrete several factors that regulate neurovascular homeostasis and stress response. Endothelial cells produce vascular endothelial growth factors and their receptors, which are expressed not only in vascular compartments, but also in neurons, neural stem cells, and microglia, facilitating neurogenesis and regenerative processes (49). Neuropilin-1, a co-receptor expressed on endothelial cells, modulates angiogenesis and inflammation by regulating transforming growth factor β signaling and endothelial responses to shear stress, thereby influencing vascular stability and immune modulation (50). The endothelial secretome encompasses cytokines and chemokines that orchestrate immune cell recruitment and activation, endothelial-derived CCL17 activates astrocytic C3 production, promoting complement-mediated microglial phagocytosis and neuroprotection against environmental nanoparticle-induced injury (51). In diabetic retinopathy, disruption of the blood-retinal barrier involves the secretion of altered vasoactive mediators by glial and vascular cells, leading to increased permeability and immune cell infiltration (52).

Collectively, the secretomes of glial, immune, and vascular cells comprise neurotrophic factors, cytokines, chemokines, ECM components, and EVs that coordinate cellular crosstalk within the NVU. This complex signaling milieu maintains CNS homeostasis under physiological conditions and mediates adaptive or maladaptive responses following ischemic injury. Understanding the regulatory roles of these cell populations provides critical insights into the mechanisms underlying neurovascular-immune interactions.

### 2.4 Structural and functional reserve: assessment of the pre-morbid and post-morbid neural architecture

Beyond the acute injury cascade, the pre-morbid and post-morbid structural status of the neural tissue profoundly modulates cognitive recovery trajectories. The microvascular reserve, defined as the density and functional capacity of the cerebral capillary network, determines the brain’s ability to maintain perfusion during ischemic stress. Relevant assessment tools include 7 T MRI angiography for capillary-level visualization, optical coherence tomography angiography (OCTA) for retinal microvascular surrogate assessment, and shear wave elastography (SWE) for pericyte-mediated vascular stiffness. Reduced microvascular reserve, commonly found in patients with chronic hypertension and diabetes, predicts both acute infarct expansion and long-term cognitive decline.

White matter integrity represents another critical reserve parameter. Diffusion tensor imaging (DTI) parameters, including fractional anisotropy (FA), mean diffusivity (MD), axial diffusivity (AD), and radial diffusivity (RD), enable the quantitative assessment of axonal and myelin integrity. Diffusion kurtosis imaging (DKI) provides additional sensitivity to the assessment of microstructural complexity beyond simpler Gaussian diffusion models (53). Myelin water fraction (MWF) mapping directly quantifies myelin content. Longitudinal DTI studies have demonstrated that pre-stroke white matter integrity, particularly in the corona radiata, superior longitudinal fasciculus, and corpus callosum, independently predicts the severity of PSCI at 6/12 months post-stroke.

Iron deposition and neuroinflammation can be quantified using advanced imaging techniques. For example, susceptibility-weighted imaging (SWI) and quantitative susceptibility mapping (QSM) can detect iron accumulation in the deep gray matter and peri-infarct regions, which correlates with cognitive impairment via ferroptosis and oxidative stress pathways (54–56). Neuromelanin-sensitive MRI assesses neuromelanin-iron complexes in the substantia nigra and locus coeruleus. Positron emission tomography (PET) tracers, including 18F-PBR111 and 11C-PK11195, target the 18 kDa translocator protein (TSPO) to visualize neuroinflammation, while 18F-FDG measures metabolic activity. Iron-sensitive PET tracers are emerging as research tools. These multimodal imaging parameters enable the comprehensive assessment of both pre-morbid reserve and post-morbid injury burden, thereby facilitating individualized prognostication and therapeutic targeting (Table 1).

**Table 1:** Cell types of the neuroecosystem, their pathological changes after cerebral ischemia–reperfusion injury, their roles in post-stroke cognitive impairment, and the associated candidate biomarkers.

Cell type	Pathological change in CIRI	Role in PSCI	Key biomarkers
Neurons	Excitotoxicity, ferroptosis, apoptosis, synaptic loss, dendritic spine instability	Information processing deficits, memory impairment, network disconnection	NfL, NfH, p-tau, SNAP29, PSD95, synaptophysin
Endothelial cells	Tight junction disruption (claudin-5 loss), MMP activation, NO reduction, oxidative stress	BBB breakdown, hypoperfusion, leukocyte infiltration	MMP-9, vWF, sICAM-1, claudin-5 (CSF)
Pericytes	Detachment from basement membrane, contraction, loss, phenotypic transition	Microvascular no-reflow, BBB instability, impaired neurovascular coupling	PDGFRbeta, soluble pericyte markers, SWE vascular stiffness
Astrocytes	AQP4 depolarization, reactive gliosis (A1/A2 skew), endfeet swelling, glycogen depletion	Glymphatic impairment, edema formation, metabolic uncoupling, synaptic dysfunction	GFAP, S100B, AQP4 polarization index
Microglia	Overactivation, polarization imbalance (simplified M1/M2), excessive synaptic pruning, DAM phenotype	Neuroinflammation, synaptic loss, tissue damage vs. repair modulation	TSPO (PET), Iba1, sTREM2, cytokine profiles
Oligodendrocytes	Demyelination, GSDMD-mediated pyroptosis, metabolic uncoupling with axons, OPC differentiation failure	White matter damage, conduction slowing, network inefficiency	MBP, NfH, myelin water fraction (MWF), DTI-FA
Neutrophils/monocytes	BBB infiltration, NET formation, M1 macrophage skew, reactive oxygen species release	Microvascular thrombosis, sustained inflammation, secondary tissue injury	cfDNA, MPO, CD14/CD16 subsets, neutrophil-lymphocyte ratio

AQP4, aquaporin-4, BBB, blood–brain barrier, cfDNA, cell-free DNA, CIRI, cerebral ischemia–reperfusion injury, CSF, cerebrospinal fluid, DAM, disease-associated microglia, DTI-FA, diffusion tensor imaging fractional anisotropy, GFAP, glial fibrillary acidic protein, GSDMD, gasdermin D, Iba1, ionized calcium-binding adapter molecule 1, MBP, myelin basic protein, MMP, matrix metalloproteinase, MPO, myeloperoxidase, MWF, myelin water fraction, NET, neutrophil extracellular trap, NfH, neurofilament heavy chain, NfL, neurofilament light chain, NO, nitric oxide, OPC, oligodendrocyte precursor cell, PDGFRβ, platelet-derived growth factor receptor β, PET, positron emission tomography, PSCI, post-stroke cognitive impairment, PSD95, postsynaptic density protein 95, p-tau, phosphorylated tau, sICAM-1, soluble intercellular adhesion molecule 1, SNAP29, synaptosome-associated protein 29, S100B, S100 calcium-binding protein B, sTREM2, soluble triggering receptor expressed on myeloid cells 2, SWE, shear wave elastography, TSPO, translocator protein, vWF, von Willebrand factor.

Table 1 summarizes the major cell types present in the neuroecosystem, their pathological changes following CIRI, and their respective roles in PSCI pathogenesis.

## 3 Deconstruction of key pathological mechanisms from the neuroecosystem perspective

### 3.1 Temporal cascade of neuroecosystem collapse: from oxidative stress to cognitive deficit

To enhance reader accessibility by clarifying the dynamic interplay among pathological effectors across disease stages, we present a temporal cascade framework linking each pathological step to the corresponding biomarkers and therapeutic interventions. This framework, summarized in Table 2, functions as a roadmap for the subsequent mechanistic sections.

**Table 2:** Temporal staging of neuroecosystem collapse after ischemic stroke, showing the dominant pathological event, key effectors, candidate biomarkers with their real-time feasibility, and the corresponding therapeutic targets at each stage.

Stage	Pathological event	Key effectors	Biomarkers (real-time feasibility)	Therapeutic targets
Hyperacute (0–6 h)	Oxidative burst, DAMPs release, PRR activation	ROS, HMGB1, ATP, TLR2/4, NLRP3 priming	Serum MDA, SOD, HMGB1 (ELISA, feasible)	Antioxidants (edaravone), DAMP scavengers, TLR antagonists
Acute (6–24 h)	Inflammasome activation, BBB disruption, leukocyte infiltration, NET formation	NLRP3 inflammasome, MMP-9, neutrophils, NETs, pericyte contraction	CSF IL-1β, MMP-9, plasma cfDNA, MPO (multiplex, feasible)	NLRP3 inhibitors, MMP inhibitors, DNase I (NETs), pericyte protectors
Subacute (24 h–7 d)	M1/M2 macrophage dynamics, microvascular thrombosis, no-reflow, astrogliosis	Monocytes, M1/M2 macrophages, reactive astrocytes, ECM remodeling	Peripheral monocyte subsets (flow cytometry), GFAP, S100B, MRI perfusion	Polarization modulators, glial modulators, ECM stabilizers
Chronic (7 d–6 mo+)	White matter degeneration, synaptic loss vs. remodeling, glymphatic impairment, cognitive network disruption	Oligodendrocytes, astrocytes, microglia, AQP4, synaptic proteins	NfH, NfL, GFAP, DTI-FA, PET (TSPO/FDG), fMRI connectivity	Remyelination agents, tDCS, sleep optimization, glymphatic enhancers, rehabilitation

ATP, adenosine triphosphate, BBB, blood–brain barrier, cfDNA, cell-free DNA, CSF, cerebrospinal fluid, DAMP, damage-associated molecular pattern, DTI-FA, diffusion tensor imaging fractional anisotropy, ECM, extracellular matrix, ELISA, enzyme-linked immunosorbent assay, FDG, fluorodeoxyglucose, fMRI, functional magnetic resonance imaging, GFAP, glial fibrillary acidic protein, HMGB1, high-mobility group box 1, IL-1β, interleukin-1β, MDA, malondialdehyde, MMP-9, matrix metalloproteinase 9, MPO, myeloperoxidase, MRI, magnetic resonance imaging, NET, neutrophil extracellular trap, NfH, neurofilament heavy chain, NfL, neurofilament light chain, NLRP3, NOD-like receptor pyrin domain-containing 3, PET, positron emission tomography, PRR, pattern recognition receptor, ROS, reactive oxygen species, SOD, superoxide dismutase, tDCS, transcranial direct current stimulation, TLR, Toll-like receptor, TSPO, translocator protein.

Immune-mediated microvascular injury differs significantly across severity grades. In mild stroke (NIHSS <5), pathology is dominated by local microglial activation, with minimal BBB disruption, reflected in mild GFAP elevation and normal neurofilament levels (57). Conversely, moderate stroke (NIHSS 5–15) features more significant neutrophil infiltration, MMP-9-mediated BBB opening, and pericyte contraction, with elevated levels of MMP-9, IL-6, and S100B (58). Severe stroke (NIHSS >15) involves extensive leukocyte infiltration, NET formation, microvascular thrombosis with no-reflow phenomenon, and high hemorrhagic transformation risk, characterized by markedly elevated cfDNA/NETs, D-dimer, ferritin, and NfH (59, 60). Assessment should integrate multiparametric MRI (PWI/DWI mismatch, hemorrhagic transformation grading), lumbar puncture for cerebrospinal fluid (CSF) cytokine profiling, multiplex serum biomarker panels, and flow cytometry for immune cell subset analysis. The following sections (3.2–3.7) deconstruct each of these core pathological mechanisms within this temporal framework.

The following four core pathological processes were selected for this detailed deconstruction because they represent cross-compartment mechanisms spanning the NIG triad, meaning they cannot be attributed to a single cell type: ferroptosis, neuroinflammation, oligodendrocyte-axon dysfunction, and gut-brain axis dysregulation. In contrast, oxidative stress and mitochondrial dysfunction function as upstream drivers of ferroptosis and neuroinflammation, BBB disruption and endothelial dysfunction are structural consequences enabling immune infiltration, and apoptosis/pyroptosis represent the downstream cell death modalities overlapping with ferroptosis. These additional mechanisms are integrated as connecting nodes in Figure 2 and are discussed within the relevant cross-compartment sections.

**Figure 2:**
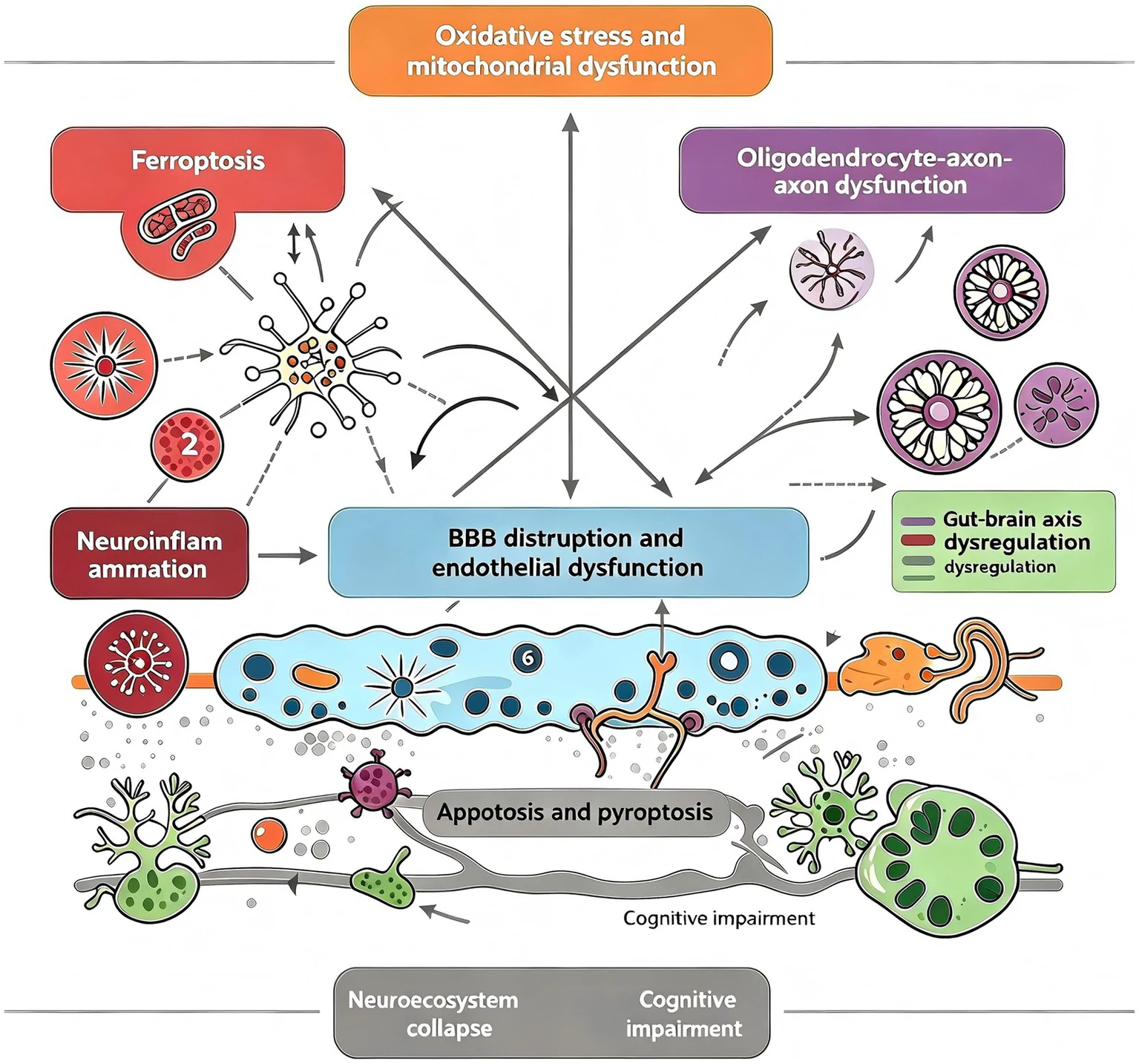
Core pathological mechanisms driving neuroecosystem collapse and post-stroke cognitive impairment after ischemic stroke. Schematic diagram illustrating the core pathological mechanisms contributing to brain neuroecosystem collapse and subsequent post-stroke cognitive impairment (PSCI). Multiple interactive pathological pathways are integrated in this model, including oxidative stress, mitochondrial dysfunction, ferroptosis, neuroinflammation, oligodendrocyte-axon dysfunction, gut-brain axis dysregulation, blood–brain barrier (BBB) disruption, endothelial dysfunction, as well as apoptosis and pyroptosis. Notably, four representative pathways (ferroptosis, neuroinflammation, oligodendrocyte-axon dysfunction, and gut-brain axis dysregulation) were selected for in-depth discussion in the manuscript, while additional critical mechanisms complement the holistic pathological network. These cascaded pathological alterations ultimately lead to neuroecosystem collapse and the occurrence of PSCI.

### 3.2 Ferroptosis and collapse of the iron metabolism cycle

Ferroptosis is a recently characterized form of regulated cell death that is strictly dependent on iron and is distinguished by lipid peroxide accumulation, reflecting a profound imbalance in iron metabolism (61, 62). In the context of CIRI, ferroptosis is a critical pathological mechanism driven by disrupted iron homeostasis within the NIG ecosystem. Iron ions, which are essential for numerous cellular functions, become deleterious when their intracellular levels are dysregulated, catalyzing reactive oxygen species (ROS) formation via Fenton chemistry, which in turn triggers oxidative stress and the lipid peroxidation of cell membranes (63). This cascade leads to cellular membrane damage and, ultimately, ferroptotic cell death. Notably, ferroptosis is not confined to neurons, it extends to glial cells such as astrocytes and microglia, as well as vascular endothelial cells, thereby compromising the integrity of the entire NVU (64). For example, ischemia/reperfusion-induced iron overload in neurons results in mitochondrial dysfunction and lipid peroxidation, contributing to cognitive deficits, whereas in astrocytes, iron accumulation promotes reactive gliosis and inflammatory responses that exacerbate neuronal injury (64).

The interplay between iron metabolism disruption and ferroptosis is further complicated by regulatory proteins, such as transferrin receptor 1, divalent metal transporter 1, ferritin, and ferroportin, whose expression levels are altered during ischemic injury, increasing iron import and impairing export. In addition, ferroptosis is modulated by key enzymes, such as acyl-CoA synthetase long-chain family member 4 and glutathione peroxidase 4 (GPX4). The downregulation of GPX4 diminishes cellular antioxidant defenses (65). Therapeutic interventions targeting ferroptosis, such as ferrostatin-1 and deferoxamine (60), and natural compounds such as calycosin and puerarin (66), have demonstrated neuroprotective effects by restoring iron balance and reducing lipid peroxidation. Non-pharmacological approaches, such as electroacupuncture and hyperbaric oxygen therapy, can also inhibit ferroptosis by modulating iron metabolism and oxidative stress markers (67).

The collapse of the iron metabolism cycle during I/R injury precipitates ferroptosis and subsequent neuronal death. Understanding the molecular underpinnings of ferroptosis across different cell types further offers promising avenues for developing targeted therapies to preserve brain function after ischemic injuries (68).

### 3.3 Neuroinflammation: dysregulated immune-glial communication and malignant reprogramming

Neuroinflammation following CIRI is mediated by the complex interplay between immune and glial cells, which play dual roles in neuroprotection and neurotoxicity (69, 70). Microglia and astrocytes rapidly respond to ischemic injuries and shift between protective and destructive phenotypes. Persistent or dysregulated activation leads to a maladaptive inflammatory response and tissue damage. This malignant reprogramming describes the transition of glial cells from a reparative state to a pro-inflammatory state (71).

Activated microglia release pro-inflammatory cytokines, such as TNF-α and IL-1β, which further stimulate astrocytes, thus forming a vicious inflammatory cycle. Damage to the BBB allows peripheral immune cells to infiltrate brain tissue and aggravate local inflammation (72). The EVs secreted by glial cells act as important mediators of intercellular communication and amplify inflammatory signals (73). Circadian dysregulation of microglia also contributes to sustained neuroinflammation (74). Therapeutic strategies targeting immune-glial crosstalk, microglial polarization, and peripheral immune infiltration can alleviate neuroinflammation and improve outcomes after CIRI (75, 76).

It is important to note that the traditional M1 (pro-inflammatory)/M2 (anti-inflammatory) dichotomy represents an oversimplification of microglial activation states. Recent single-cell RNA sequencing and spatial transcriptomics studies have identified a continuum of microglial phenotypes, which do not fit neatly into the M1/M2 binary, including disease-associated microglia (DAM), interferon-responsive microglia, and proliferative-region-associated microglia (PAM) (77, 78). Furthermore, microglial phenotypes are highly dynamic and context-dependent, transitioning between states in response to temporal, spatial, and metabolic cues. In this review, we use M1/M2 terminology for conceptual clarity, while acknowledging that the actual *in vivo* situation involves a spectrum of activation states.

### 3.4 White matter injury: oligodendrocyte-axon metabolic uncoupling

Oligodendrocytes form myelin sheaths for axons and maintain close metabolic coupling with axons to support long-distance neural conduction. CIRI induces oligodendrocyte damage, demyelination, and axonal degeneration, resulting in white matter injury and disrupted information transmission.

Gasdermin D (GSDMD)-mediated pyroptosis in oligodendrocytes exacerbates demyelination and axonal injury (79). The failure of oligodendrocyte-axon metabolic coupling leads to axonal energy deficiency and accelerates neural circuit dysfunction, which is a key driver of PSCI. Targeting inflammasome and GSDMD pathways helps to protect white matter integrity and cognitive function (79).

Axonal damage and active degeneration can be monitored using biomarkers, including neurofilament heavy chain (NfH), neurofilament light chain (NfL), phosphorylated tau (p-tau), and beta-amyloid precursor protein (beta-APP) accumulation in axonal swellings. These markers distinguish active Wallerian degeneration from chronic stable white matter lesions. Synaptic loss versus remodeling is a critical determinant of cognitive outcome: postsynaptic density protein 95 (PSD95), synaptophysin, and SNAP29 levels quantify synaptic density, while brain-derived neurotrophic factor (BDNF) and its receptor TrkB reflect activity-dependent synaptic plasticity. The balance between synaptic pruning (mediated by complement C1q/C3 and microglial phagocytosis) and compensatory synaptogenesis determines whether cognitive networks undergo functional reorganization or progressive degeneration.

### 3.5 Gut-brain axis: impact of systemic exogenous injuries on the neuroecosystem

The gut-brain axis forms a bidirectional communication pathway between the intestinal microbiota and the CNS. CIRI induces gut dysbiosis, disrupts intestinal barrier function, and triggers systemic inflammation (80, 81). Microbial metabolites and inflammatory factors enter the brain through the circulation and aggravate neuroinflammation and ferroptosis, thereby forming a systemic vicious cycle.

Interventions such as probiotics, prebiotics, and fecal microbiota transplantation can all help to regulate the gut flora, reduce systemic inflammation, and indirectly protect the brain neuroecosystem (80, 81). Environmental pollutants, such as fine particulate matter, also worsen brain injury via the gut-brain axis (81). This systemic regulatory pathway is an important new target for the treatment of CIRI and PSCI.

### 3.6 Organelle and metabolic dysfunction: mitochondria, proteostasis, and autophagy

Beyond the four core cross-compartment mechanisms, organelle-level dysfunction constitutes a fundamental driver of neuroecosystem collapse and warrants detailed examination. In particular, mitochondrial dysfunction serves as a central hub connecting oxidative stress, ferroptosis, neuroinflammation, and energy failure. During CIRI, mitochondrial fission-fusion imbalance occurs through dynamin-related protein 1 (Drp1)-mediated excessive fission and mitofusin 2 (Mfn2) downregulation, leading to mitochondrial fragmentation (82–84). The PINK1/Parkin pathway mediates mitophagy, which is impaired following reperfusion, resulting in the accumulation of damaged mitochondria, mtDNA damage, ROS overproduction, and ATP depletion (85). The mitochondrial permeability transition pore (mPTP) opening triggers cytochrome c release and apoptosis. Notably, intercellular mitochondrial transfer from astrocytes to neurons via tunneling nanotubes and EVs represents an endogenous repair mechanism that becomes compromised in severe CIRI.

Proteostasis impairment further exacerbates cellular injury. Notably, the ubiquitin-proteasome system is inhibited by oxidative stress, leading to the accumulation of misfolded and ubiquitinated proteins that form aggresomes. The heat shock protein response, including HSP70 and HSP90, is activated as a compensatory mechanism, but is frequently overwhelmed in severe ischemia (86). Autophagy dysregulation plays a dual role: in the acute phase, excessive autophagy (characterized by an increased LC3-II/I ratio, Beclin-1 upregulation, and p62/SQSTM1 degradation) can promote autophagic cell death, in the subacute phase, impaired autophagic flux (defined as accumulation of autophagosomes with impaired lysosomal degradation) prevents the clearance of damaged organelles and protein aggregates. The integrated stress response, which is mediated by eIF2alpha phosphorylation and ATF4 activation, modulates the balance between pro-survival adaptation and pro-death signaling. Monitoring these organelle pathways through transmission electron microscopy, Seahorse extracellular flux analysis, and immunoblotting of LC3, p62, Drp1, Mfn2, and PINK1 provides mechanistic insights beyond conventional cell death markers.

### 3.7 Glymphatic dysfunction and sleep–wake cycle disruption

The glymphatic system, a perivascular network that facilitates cerebrospinal fluid influx and interstitial fluid (ISF) clearance, plays an essential role in maintaining neuroecosystem homeostasis. Anatomically, glymphatic function depends on the polarized expression of aquaporin-4 (AQP4) on astrocytic perivascular endfeet, which drives the convective flow of ISF along the perivascular spaces. This system clears metabolic waste, including amyloid-beta, tau, lactate, and inflammatory mediators, from the brain parenchyma. Following CIRI, AQP4 depolarization, perivascular space enlargement, and astrocytic endfeet swelling collectively impair glymphatic clearance, resulting in the accumulation of neurotoxic solutes and sustained neuroinflammation that accelerates cognitive decline (87).

Sleep–wake cycle regulation is intrinsically coupled to glymphatic function. Indeed, glymphatic flux increases by approximately 60% during non-rapid eye movement (NREM) sleep, driven by a reduction in noradrenergic tone that expands the extracellular space and enhances convective flow. Up to 70% of stroke survivors are affected by post-stroke sleep architecture disruption, including insomnia, sleep-disordered breathing (obstructive sleep apnea), circadian misalignment, and excessive daytime sleepiness, this disruption significantly impairs glymphatic clearance during critical recovery periods. Sleep deprivation reduces synaptic plasticity-related protein expression (BDNF and PSD95) and increases pro-inflammatory cytokine levels, thereby creating a vicious cycle of glymphatic impairment, neuroinflammation, and cognitive deterioration.

Assessment of glymphatic function includes MRI-based perivascular space (PVS) scoring, DTI analysis along the perivascular space (DTI-ALPS), and intrathecal contrast-enhanced MRI glymphatic imaging. Sleep assessment employs polysomnography (gold standard), actigraphy, and validated questionnaires, including the Pittsburgh Sleep Quality Index (PSQI) and Insomnia Severity Index (ISI). Therapeutic interventions targeting the glymphatic-sleep axis include continuous positive airway pressure for obstructive sleep apnea, circadian light therapy, melatonin supplementation, AQP4 modulators, and structured physical exercise, which reportedly enhances glymphatic clearance and improves cognitive outcomes in preclinical stroke models.

In the long-term phase, the gut microbiome engages in a complex crosstalk with adipose tissue, neutrophils, and obesity-related pathways that modulate cognitive recovery. Microbial metabolites, including short-chain fatty acids (SCFAs), lipopolysaccharide (LPS), and trimethylamine N-oxide (TMAO), can modulate adipocyte function and systemic metabolic homeostasis. Visceral obesity drives chronic low-grade inflammation through dysregulated adipokine secretion, including elevated leptin and resistin and reduced adiponectin, which synergize with gut-derived inflammatory signals to accelerate cognitive decline. The gut-neutrophil axis represents another critical pathway: microbiota-derived signals prime neutrophil activation and neutrophil extracellular trap (NET) formation, while gut dysbiosis-induced systemic inflammation amplifies neutrophil-mediated microvascular injury in the brain. The cognitive domains affected by these systemic insults are determined by regional brain vulnerability, particularly in the hippocampus, prefrontal cortex, and periventricular white matter. Long-term interventions include dietary modulation (such as the Mediterranean diet and time-restricted feeding), probiotics and prebiotics, exercise-induced microbiome remodeling, and bariatric surgery, all of which have shown preliminary evidence of cognitive benefits through gut-brain axis modulation.

Figure 2 illustrates the interconnected nature of key pathological drivers of PSCI. Four interacting core pathological processes form a vicious cycle: ferroptosis and metabolic collapse, maladaptive neuroinflammation, oligodendrocyte-axon unit failure, and gut-brain axis dysregulation. The bidirectional interactions between these pathways ultimately lead to neuroecosystem collapse and cognitive deficits.

## 4 Application of advanced technologies in neuroecosystem research

### 4.1 Spatial omics technologies reveal intercellular spatial associations

Single-cell spatial transcriptomics and proteomics technologies have enabled researchers to analyze the molecular profiles and spatial distribution of cells in intact brain tissue (88, 89). Ischemic injury induces the spatial remodeling of neurons, glia, and immune cells, which can be accurately mapped by spatial omics (90). Integrated spatial multiomics further reveals metabolic changes and cell–cell crosstalk in pathological microenvironments (91). Further, three-dimensional visualization tools and simulation algorithms facilitate the analysis of complex spatial data and cell interaction networks (92, 93). Spatial omics provides powerful technical support for exploring the spatial dynamics of the brain neuroecosystem after injury.

Specifically in ischemic stroke research, 10x Genomics Visium spatial transcriptomics has been applied to middle cerebral artery occlusion (MCAO) mouse brains, revealing distinct peri-infarct reactive astrocyte zones characterized by the upregulation of *Lcn2*, *Serpina3n*, and *Gfap*, as well as microglial activation gradients extending from the infarct core to the contralateral hemisphere (94, 95). Spatial proteomics techniques, including CODEX (co-detection by indexing) and multiplexed ion beam imaging (MIBI), have mapped the immune cell infiltration patterns in postmortem human stroke tissue, identifying neutrophil-rich perivascular cuffs and lymphocyte clusters in the peri-infarct region. Spatial metabolomics using matrix-assisted laser desorption/ionization mass spectrometry imaging (MALDI-MSI) has mapped ATP, glutamate, and lipid peroxide distributions in the ischemic penumbra, correlating metabolic failure with ferroptosis susceptibility. Slide-seq and MERFISH (multiplexed error-robust fluorescence *in situ* hybridization) studies have further revealed synaptic gene expression gradients in the post-stroke hippocampus linked to cognitive deficits, particularly in genes regulating NMDA receptor trafficking and dendritic spine morphology.

### 4.2 *In vivo* imaging techniques monitoring functional coupling dynamics

*In vivo* imaging technologies such as multiphoton microscopy and functional MRI (fMRI) enable real-time observation of neural network activity and vascular dynamics in living animals (96, 97). Two-photon imaging can track synaptic and microglial dynamic changes following brain injury (98). Dynamic functional connectivity analysis of fMRI reflects network activity fluctuations (97). Combined with calcium imaging and molecular labeling, these techniques can be used to quantify cellular activity and signal transduction (99). Multimodal *in vivo* imaging is critical for revealing the dynamic coupling and functional reconstruction of the NIG triad post-CIRI.

In ischemic stroke and PSCI research, two-photon *in vivo* imaging through cranial windows has enabled the real-time tracking of dendritic spine dynamics in MCAO mice during cognitive task learning, revealing that the peri-infarct cortex exhibits increased spine turnover, but reduced spine stability, correlating with impaired memory consolidation (100). Multiphoton imaging has further demonstrated microglial process motility and synaptic engulfment in the peri-infarct region, showing that the excessive microglial pruning of complement-tagged synapses contributes to cognitive deficits. fMRI studies in patients with PSCI have identified disrupted default mode network connectivity, executive control network inefficiency, and altered salience network dynamics, with functional connectivity patterns predicting cognitive recovery trajectories. Arterial spin labeling MRI measures cerebral blood flow changes during rehabilitation, thereby enabling the assessment of neurovascular coupling recovery. PET imaging with TSPO tracers has additionally tracked neuroinflammation resolution in longitudinal stroke cohorts, while 18F-FDG PET has mapped the metabolic recovery patterns associated with cognitive improvement. The multimodal integration of these imaging modalities facilitates the comprehensive monitoring of neuroecosystem restoration.

## 5 A paradigm shift in therapeutic strategies

### 5.1 Limitations of single-target neuroprotection

Traditional therapies for CIRI and PSCI have primarily focused on protecting neurons alone, ignoring the overall NIG network dysfunction (101, 102). Although drugs such as morin, tangeretin, and salvianolic acid A exert partial neuroprotective effects, they cannot reverse complex network lesions involving inflammation, vascular damage, and glial activation (103, 104). Many clinical trial failures of neuroprotective drugs have been attributed to the single-target limitation, wherein a single intervention cannot address the multiple pathological processes of neuroecosystem collapse (105).

### 5.2 Ecological restoration and homeostatic reconstruction concepts

The neuroecosystem restoration strategy aims to comprehensively repair the NVU, neuronal networks, and the glial-immune microenvironment to re-establish brain homeostasis. It involves stabilizing BBB function, regulating glial and immune phenotypes, and promoting neural plasticity (106). Circadian rhythm regulation is further involved in post-stroke repair processes. Timed intervention combined with circadian rules can improve therapeutic efficacy (106). This holistic treatment paradigm is fundamentally distinct from single-target neuroprotection.

### 5.3 Combined therapies, sequential therapies, and systemic intervention strategies

Given the complexity of CIRI pathology, combined multi-target therapy, staged sequential therapy, and systemic intervention have all become increasingly recognized directions (107, 108). In the acute phase, treatment focuses on protecting NVU and inhibiting acute cell death such as ferroptosis. In the subacute phase, treatment mainly aims to regulate neuroinflammation and promote tissue repair. Finally, in the chronic phase, treatment emphasizes remyelination, neural plasticity, and systemic regulation (such as gut microbiota modulation). Systemic interventions targeting the gut-brain axis and peripheral immunity work together with intracranial treatments to achieve overall neuroecosystem repair (109, 110).

### 5.4 Biomarkers of resilience and recovery: beyond the M1/M2 paradigm

Monitoring the mechanisms underlying resilience and cellular recovery requires biomarkers that extend beyond the traditional M1/M2 microglial classification and astroglial activation markers. Protein translation signatures offer mechanistic resolution: polysome profiling and ribosome sequencing (Ribo-seq) can distinguish pro-death from pro-repair translational programs, with the mTORC1/eIF2alpha/ATF4 integrated stress response functioning as a critical node. Increased translation of plasticity-related transcripts (such as BDNF, Arc, Homer1, activity-regulated cytoskeleton-associated protein) indicates active synaptic remodeling, while the sustained translation of pro-inflammatory and pro-apoptotic transcripts suggests ongoing damage.

Structural plasticity markers include growth-associated protein-43 (GAP-43), microtubule-associated protein-2 (MAP-2), and the PSD95/synaptophysin ratio, which together distinguish compensatory synaptogenesis from net synaptic loss (111). Functional plasticity can further be assessed through long-term potentiation (LTP) recordings in hippocampal slices, resting-state fMRI functional connectivity, and network efficiency metrics. EV cargo provides minimally invasive biomarkers of resilience: serum/plasma EVs carrying miR-21, miR-124, BDNF, and IGF-1 all reflect glial-neuronal protective signaling, while EV-associated pro-inflammatory miRNAs (miR-155, miR-146a) indicate sustained neuroinflammation (112, 113). Epigenetic recovery markers, including BDNF promoter DNA methylation status, histone acetylation patterns (H3K9ac, H3K27ac), and circulating non-coding RNA profiles, enable the longitudinal monitoring of recovery programs. Integrating these multi-level biomarkers with conventional clinical assessments would enable the precise evaluation of neuroecosystem restoration and prediction of long-term cognitive outcomes.

Figure 3 divides the post-stroke process into the acute, subacute, and chronic phases, and matches targeted interventions at systemic, vascular, cellular, and molecular levels. Sequential and combined therapies are adopted to achieve restoration of the neuroecosystem.

**Figure 3:**
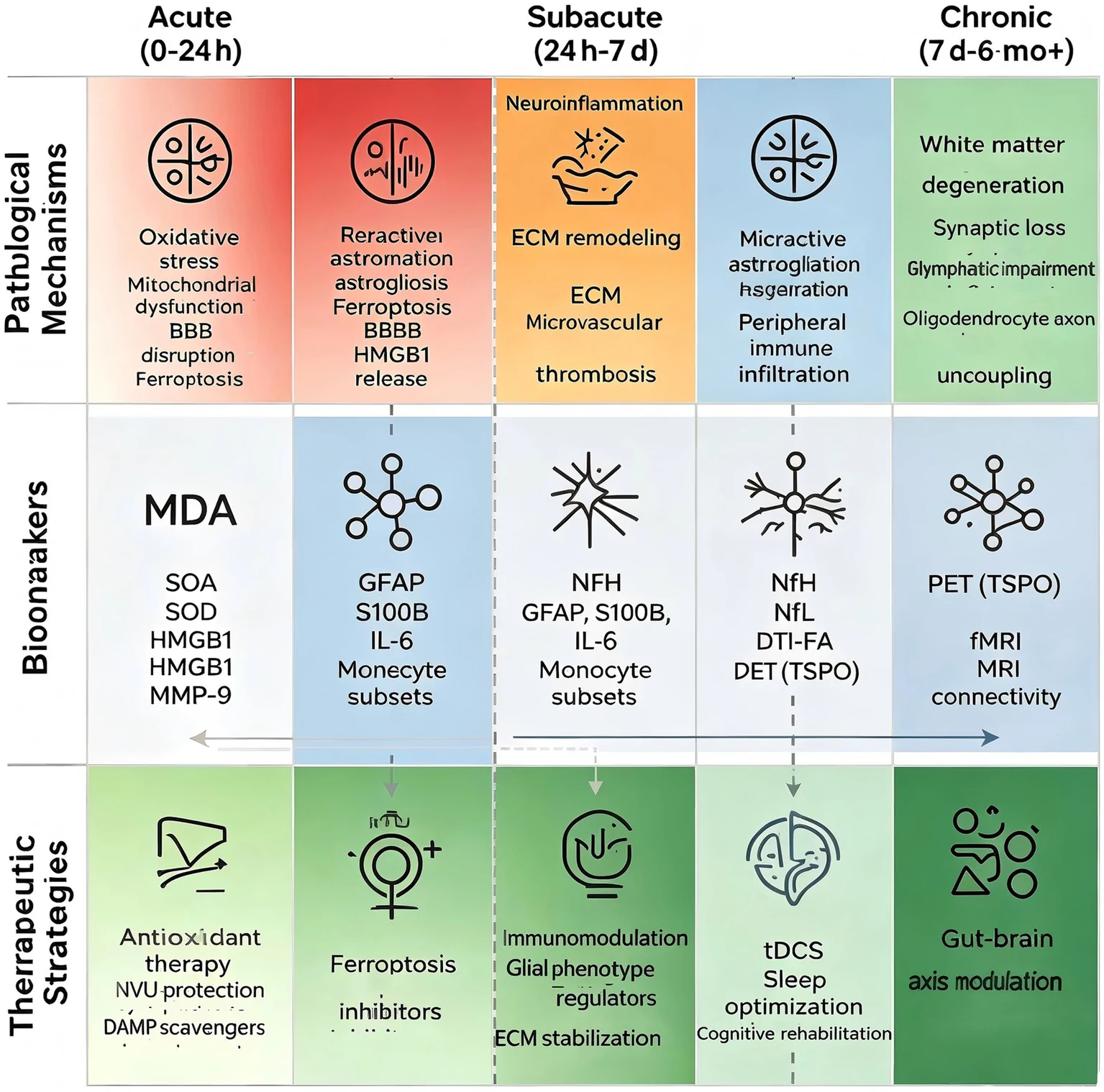
Temporal profiles of pathological mechanisms, biomarkers and therapeutic strategies across acute, subacute and chronic phases after ischemic stroke. Temporal overview of pathological cascades, detectable biomarkers and phase-targeted therapeutic strategies following ischemic stroke. Pathological events evolve dynamically from the acute (0–24 h), subacute (24 h–7 d) to chronic (7 d–6 mo+) stages. Representative circulating and neuroimaging biomarkers are listed for each time window. Corresponding therapeutic interventions are summarized according to phase-specific pathological features to guide staged management for post-stroke brain injury.

## 6 Future prospects and key scientific issues

### 6.1 Testable hypothesis and experimental design

The core hypothesis of this review is that PSCI is essentially driven by the collapse of the NIG-based brain neuroecosystem, in which multiple pathological pathways interact to drive disease progression. Multiple animal models, such as the MCAO model, can be used for verification (114). Experiments will need to be conducted to detect ferroptosis, inflammation, glial activation, and other indicators, combined with behavioral tests to evaluate cognitive function (115, 116). Clarifying the crosstalk between ferroptosis and neuroinflammation will be a key research direction (117, 118).

### 6.2 New technologies driving mechanistic research and clinical translation

Spatial omics and dynamic imaging need to be combined to achieve the long-term tracking of neuroecosystem changes after injury (119). Developing ecosystem-specific biomarkers will be conducive to early diagnosis and efficacy evaluation (120). Novel drug delivery systems and multimodal therapies further need to be translated from basic experiments to clinical practice (121).

### 6.3 Interdisciplinary collaboration and systems biology model

Cooperation between neuroscience, immunology, microbiology, and computational biology is needed to build systems biology models of the neuroecosystem (122, 123). Artificial intelligence and big data analysis could help to identify core targets and enable precision medicine (124). Interdisciplinary research will greatly promote the theoretical and clinical progress of this field.

### 6.4 Long-term dynamic monitoring and cognitive recovery evaluation

Long-term follow-up combining behavioral assessment, imaging, and molecular detection will be required to evaluate the efficacy of repair therapies (125, 126). Studying the mechanisms underlying synaptic remodeling, epigenetic changes, and exosome communication during recovery helps to find new rehabilitation targets (127, 128).

## 7 Limitations

Several limitations of this review should be acknowledged. First, although we endeavored to prioritize stroke-specific evidence, some mechanistic data, particularly regarding glymphatic function, spatial omics, and organelle-level pathways, were extrapolated from other neurological disorders including Alzheimer’s disease, multiple sclerosis, and glaucoma, and will accordingly require direct validation in ischemic stroke models and patient cohorts. Second, the temporal staging of pathological events represents a simplified framework, in reality, the different mechanisms overlap considerably and exhibit substantial inter-individual variability based on stroke subtype, severity, comorbidities, and genetic background. Third, many of the proposed biomarkers and therapeutic strategies remain in the preclinical stages, and their clinical utility in PSCI accordingly requires prospective validation in adequately powered longitudinal cohorts. Future research should therefore prioritize stroke-specific mechanistic studies, multimodal biomarker panels integrated with clinical outcomes, and randomized controlled trials of ecosystem-level interventions.

## 8 Conclusion

In conclusion, this review highlights that an evolving understanding of PSCI necessitates a conceptual shift from the traditional neuron-centric view to a comprehensive neuroecosystem perspective. Historically, the focus on neuronal death has provided valuable insights, but is insufficient to fully explain the complex pathological characteristics underlying CIRI and PSCI. The NIG triad, consisting of vascular units, neuronal networks, and the glial-immune microenvironment, forms the core of the brain neuroecosystem. Ferroptosis, neuroinflammation, white matter injury, and gut-brain axis disorders are interactive manifestations of neuroecosystem collapse.

Therapeutically, single-target interventions have limited efficacy. The new treatment paradigm should accordingly focus on overall ecosystem homeostasis reconstruction, adopting combined, staged, and systemic strategies. Emerging technologies, such as spatial omics and *in vivo* imaging, represent powerful tools for verifying the neuroecosystem theory. Future research needs to strengthen mechanistic validation, interdisciplinary collaboration, and clinical translation.

The neuroecosystem framework provides a new holistic perspective for the study of ischemic brain injury and cognitive complications, and has important implications for the development of new diagnostic markers and therapeutic strategies in patients with stroke.
